# Dengue Outbreak during Ongoing Civil War, Taiz, Yemen

**DOI:** 10.3201/eid2507.180046

**Published:** 2019-07

**Authors:** KhairAlah A. Alghazali, Boon-Teong Teoh, Shih-Keng Loong, Sing-Sin Sam, Nurul-Asma-Anati Che-Mat-Seri, Nur-Izyan Samsudin, Che-Norainon Yaacob, Noor-Syahida Azizan, Adrian Oo, Nur-Adilah Baharudin, Kim-Kee Tan, Juraina Abd-Jamil, Siti-Sarah Nor’e, Chee-Sieng Khor, Jefree Johari, Mohammed A.K. Mahdy, Sazaly AbuBakar

**Affiliations:** University of Malaya, Kuala Lumpur, Malaysia (K.A. Alghazali, B.-T. Teoh, S.-K. Loong, S.-S. Sam, N.-A.-A. Che-Mat-Seri, N.-I. Samsudin, C.-N. Yaacob, N.-S. Azizan, A. Oo, N.-A. Baharudin, K.-K. Tan, J. Abd-Jamil, S.-S. Nor’e, C.-S. Khor, J. Johari, S. AbuBakar);; University of Science and Technology, Sana’a Yemen (M.A.K. Mahdy)

**Keywords:** dengue, dengue virus, dengue virus type 2, viruses, arbovirus, vector-borne infections, outbreak, arbovirus, conflict, displacement, civil war, zoonoses, Taiz, Yemen

## Abstract

We identified dengue in ≈51% of patients given a clinical diagnosis of suspected dengue in Taiz, Yemen, during 2016. The cosmopolitan genotype of dengue virus type 2 was most common; viruses appeared to have originated in Saudi Arabia. Damage to public health infrastructure during the ongoing civil war might enable dengue to become endemic to Yemen.

The association between wars and dengue virus (DENV) transmission has been well-recognized. During World War II (1939–1945), extensive ecologic disruption and demographic changes created an abundance of ideal breeding sites for *Aedes aegypti* mosquitoes, as well as pools of susceptible military personnel and displaced populations to support the spread of dengue (*1*). After World War II, unprecedented population growth and rapid unplanned urbanization exacerbated the epidemic spread of dengue to the major cities of Southeast Asia and the Pacific regions (*2*).

The current civil war in Yemen, which started in March 2015 (*3*), has caused widespread destruction of the infrastructure of this country and displaced >2.2 million persons into living in cramped shelters with inadequate healthcare support. The sum of human displacement, damaged infrastructure, and poor hygiene conditions (*3**–**5*) has created an ideal environment for the spread of infectious diseases, including mosquitoborne diseases. In particular, the war has created numerous potential mosquito-breeding sites, such as open water storage containers, areas with inadequate drainage, discarded plastic containers in which water accumulates, and puddles of water (*6*).

In 2015, a total of >6,777 suspected dengue cases were recorded in Yemen, which suggested that the country was experiencing an unprecedented increase in the number of dengue cases (*7*). Taiz, a governorate in southwestern Yemen, experienced fierce fighting related to the civil war (*5*). An extreme spike in dengue cases was recorded in this governorate beginning in August 2015, soon after the start of the war. A total of 1,178 suspected dengue cases were reported during weeks 32–36 (*7*) in comparison to only 54 suspected dengue cases during the same period in 2013 (*8*). We report the prevalence, detection, and isolation of DENV from febrile patients seen at the few operating healthcare facilities in Taiz during July–October 2016, at the height of the war.

## The Study

**Figure 1:**
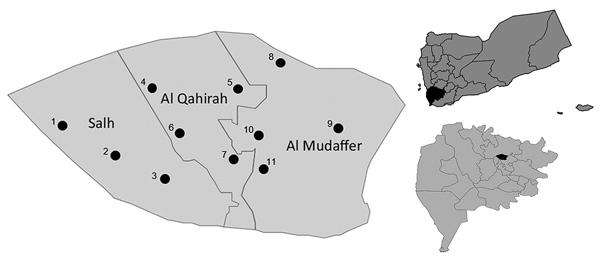
Locations of hospitals and medical centers in Taiz, Yemen, where dengue virus samples were obtained during 2016: 1, Aljawharah Medical Center; 2, Gulf Hospital; 3, Alrefaee Hospital; 4, Althawrah Hospital; 5, Altawn Hospital; 6, Alrawdhah Hospital; 7, Alsawidy Hospital; 8, Palastein Hospital; 9, Alboraihy Hospital; 10, Alhekmah Hospital; 11, Dr. Sadek Shogaa Center. Top inset shows location of Taiz in Yemen (black shading), and bottom inset shows location of collection area in Taiz (black shading).

The study was approved by the University of Malaya Science and Technology Medical Ethical Committee (approval no. 2016/24). A total of 436 serum specimens were obtained from patients with clinically suspected dengue (age range 1–70 years) who sought healthcare within 2–7 days after the onset of fever (Appendix Figure 1). These patients were seen at the main hospitals and medical centers that were still in operation (Figure 1) in Taiz during the study period. Samples were kept at −20°C until they were transported to the World Health Organization Collaborating Centre for Arbovirus Reference and Research (Dengue/Severe Dengue) at the Tropical Infectious Diseases Research and Education Centre, University of Malaya (Kuala Lumpur, Malaysia).

For detection of DENV, we extracted total RNA from serum specimens by using the KingFisher Pure Viral NA Kit (Thermo Fisher Scientific, https://www.thermofisher.com). We used a DENV-specific reverse transcription–recombinase polymerase amplification (RT-RPA) to screen for DENV RNA (*9*). We subjected all RT-RPA–reactive specimens to molecular DENV serotyping by using DengueDetect, an in-house multiplex reverse transcription PCR (RT-PCR) kit (http://www.tidrec.com/services). We tested all RT-RPA–nonreactive specimens for IgM against DENV by using the SD Dengue IgM-Capture ELISA Kit (Standard Diagnostics Inc., https://www.devex.com). All DENV IgM–nonreactive specimens were tested for DENV IgG by using the SD Dengue IgG-Capture ELISA Kit (Standard Diagnostics Inc.). All diagnostic tests were performed in an MS ISO/IEC 17025–accredited laboratory at the Tropical Infectious Diseases Research and Education Centre, University of Malaya.

**Figure 2:**
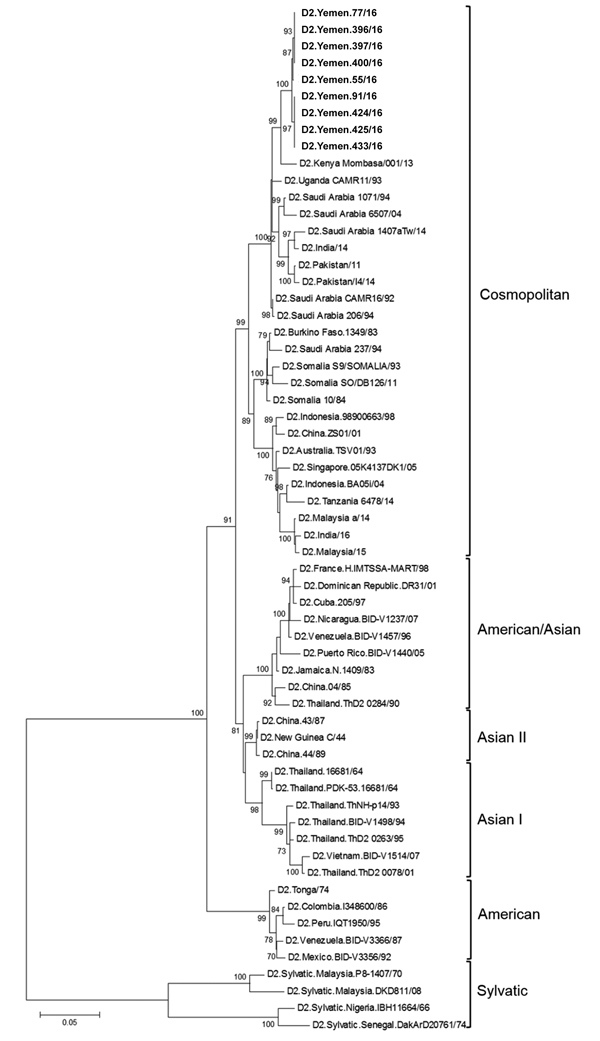
Maximum-likelihood phylogenetic tree of dengue virus type 2 isolates from Taiz, Yemen, 2016 (top branch), and reference isolates. The tree was constructed by using envelope gene sequences. Numbers on nodes indicate bootstrap values (%) for 1,000 replicates. Only bootstrap values >70% are indicated. Scale bar indicates nucleotide substitutions per site.

Of 436 patients with clinically suspected dengue, 119 (≈27%) were reactive by RT-RPA; among those patients, 83 (≈70%) were serotyped by RT-PCR as DENV-2. The remaining 36 RT-RPA–reactive specimens were nonreactive by RT-PCR. However, the RT-RPA is known to be more sensitive than the RT-PCR; thus, this result could be caused by low viral loads in the specimens (*9*). All RT-PCR–reactive specimens were further subjected to virus isolation by using the C6/36 mosquito cell line. Complete envelope (E) genes of DENV-2 were successfully amplified by using primers specific for the DENV-2 E gene (Appendix Table) and sequenced from 9 of 83 specimens after a third passage in cell culture. A phylogenetic tree constructed by using E genes suggested that the DENV-2 isolates from Yemen clustered within the DENV-2 Cosmopolitan genotype (Figure 2). The E gene sequences generated in this study are available from the European Nucleotide Archive (accession no. PRJEB27739).

Of the 317 RT-RPA–nonreactive specimens, 102 (≈32%) were positive for DENV IgM. Of 215 dengue IgM–nonreactive samples, 74 (≈34%) were positive for DENV IgG. Those samples positive for only DENV IgG were likely caused by previous exposure to DENV infection. We identified acute DENV infection in ≈51% (221 of 436) of febrile patients with suspected dengue either by RT-RPA or DENV IgM ELISA. In addition, ≈34% (74 of 215) of febrile patients who did not have dengue had previous exposure to dengue.

## Conclusions

In this study, the prevalence of dengue among patients with suspected dengue (≈51%) in Taiz was higher than that (29%) during the previous dengue outbreak in Hodeidah in 2012 (*10*). The percentage of previous exposure to DENV among febrile patients was higher in Hodeidah (≈75%) than that seen in Taiz (≈34%) (*10*). It is possible that dengue is a relatively new disease in Taiz, but its prevalence has increased markedly, possibly because of the ongoing civil war. The febrile patients who did not have dengue seen in Taiz could have had other infections, including chikungunya and malaria, because these 2 mosquitoborne infectious diseases are also present in Yemen (chikungunya prevalence 12% and malaria prevalence 15.3%) (*10**,**11*). Nonetheless, in a previous study in Al-Mukalla, 222 patients with clinically suspected viral hemorrhagic fever were nonreactive to chikungunya virus, Alkhurma virus, Rift Valley fever virus, and yellow fever virus by RT-PCR (*12*), suggesting that other studies are needed to identify the possible causative agents.

Before the current civil war began, all 4 DENV serotypes were present in Yemen, but only DENV-2 (*10*), DENV-3 (*12**,**13*), and DENV-4 (*14*) have been documented to cause outbreaks. Using partial nonstructural protein 1 gene sequences, Ciccozzi et al. identified the DENV-2 Cosmopolitan genotype as the virus that caused the outbreak in Hodeidah during 2012 (*15*). We report that this virus type was also found as the dominant virus causing the outbreak in Taiz during 2016. However, the 2012 and 2016 viruses grouped in different clades within the DENV-2 Cosmopolitan genotype (Appendix Figure 2). The origin of the viruses that caused the outbreak during 2012 is uncertain, whereas the viruses that caused the outbreak during 2016 grouped in a clade composed mainly of DENV-2 isolates (1992–2014) from Saudi Arabia. This finding suggests that the DENV-2 that caused the outbreak in Yemen during 2016 was most likely introduced from Saudi Arabia and that the ongoing civil war might ensure its lasting presence.

Dengue is emerging to be a serious mosquitoborne disease in war-torn Yemen. Its presence among 51% of febrile patients suspected to have dengue is almost comparable to the percentage reported in dengue-endemic countries of Southeast Asia. It is expected that the public health problems associated with dengue will worsen during the continuing civil war in Yemen.

Appendix Additional information on dengue outbreak during current civil war, Taiz, Yemen.
