# Supplementary material for: Dengue Outbreak during Ongoing Civil War, Taiz, Yemen
Source: Emerg Infect Dis. 2019 Jul;25(7):1397–400. doi: 10.3201/eid2507.180046 (PMC6590741; doi:10.3201/eid2507.180046)
Supplement: Appendix — Additional information on dengue outbreak during current civil war, Taiz, Yemen. [file 18-0046-Techapp-s1.pdf]

Article DOI: https://doi.org/10.3201/eid2506.180046

Dengue Outbreak during Ongoing Civil War, Taiz, Yemen

Appendix

Appendix Table. Primers used for amplification and sequencing of the DENV-2 envelope protein gene*

Primer name	Sequence, 5′→3′	Position†	Use
D2.553F	ATRGACCTTGGTGARYTGTG	553–572	Amplification and sequencing
D2.1285R	CACAGGTYACRAYRCCTCC	1267–1285	Sequencing
D2.1825R	TTCCTTTGRGCTGTARYTTGTC	1804–1825	Sequencing
D2.2075F	TAGGAGTAGARCCRGGACA	2075–2093	Sequencing
D2.2279R	AAGGCARCHCCRTAGATT	2262–2279	Sequencing
D2.2705R	CCTTTRATGTCTCCTGTCAT	2686–2705	Amplification and sequencing

*DENV-2, dengue virus type 2.
†All nucleotide positions refer to the published complete genome of DENV-2 strain 16681 (GenBank accession no. NC_001474).

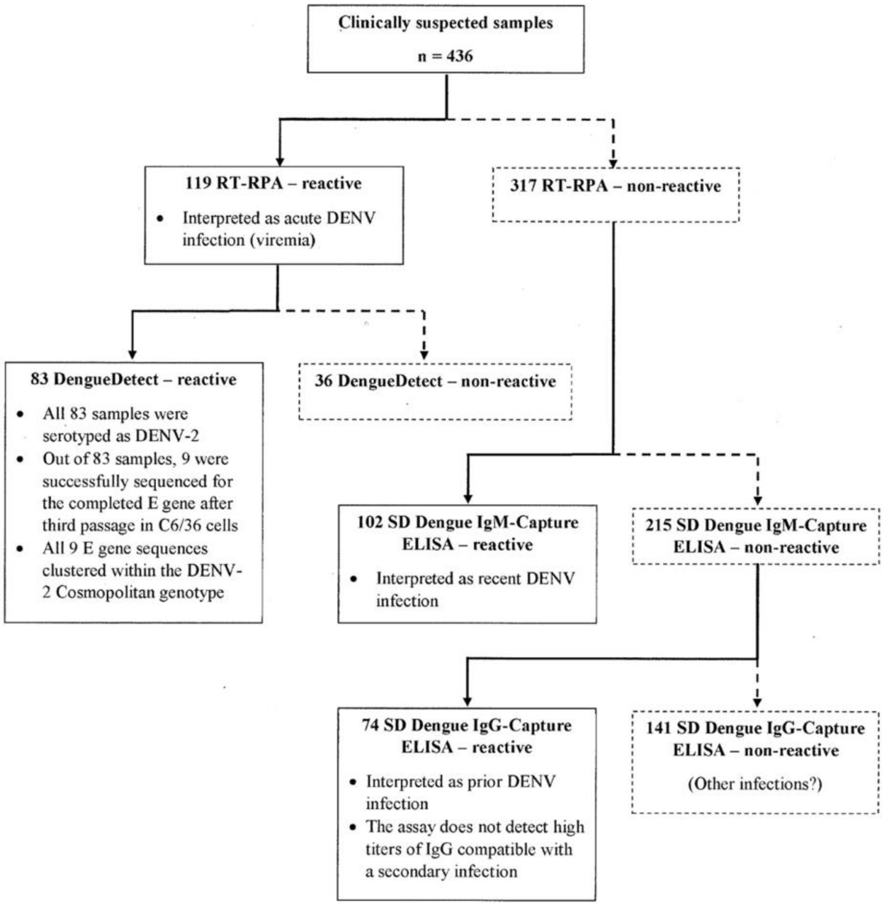

Appendix Figure 1. Flowchart detailing serologic and virologic data for patients during dengue outbreak during ongoing civil war, Taiz, Yemen. DENV-2, dengue virus type 2; E, envelope; RT-RPA, reverse transcription–recombinase polymerase amplification.

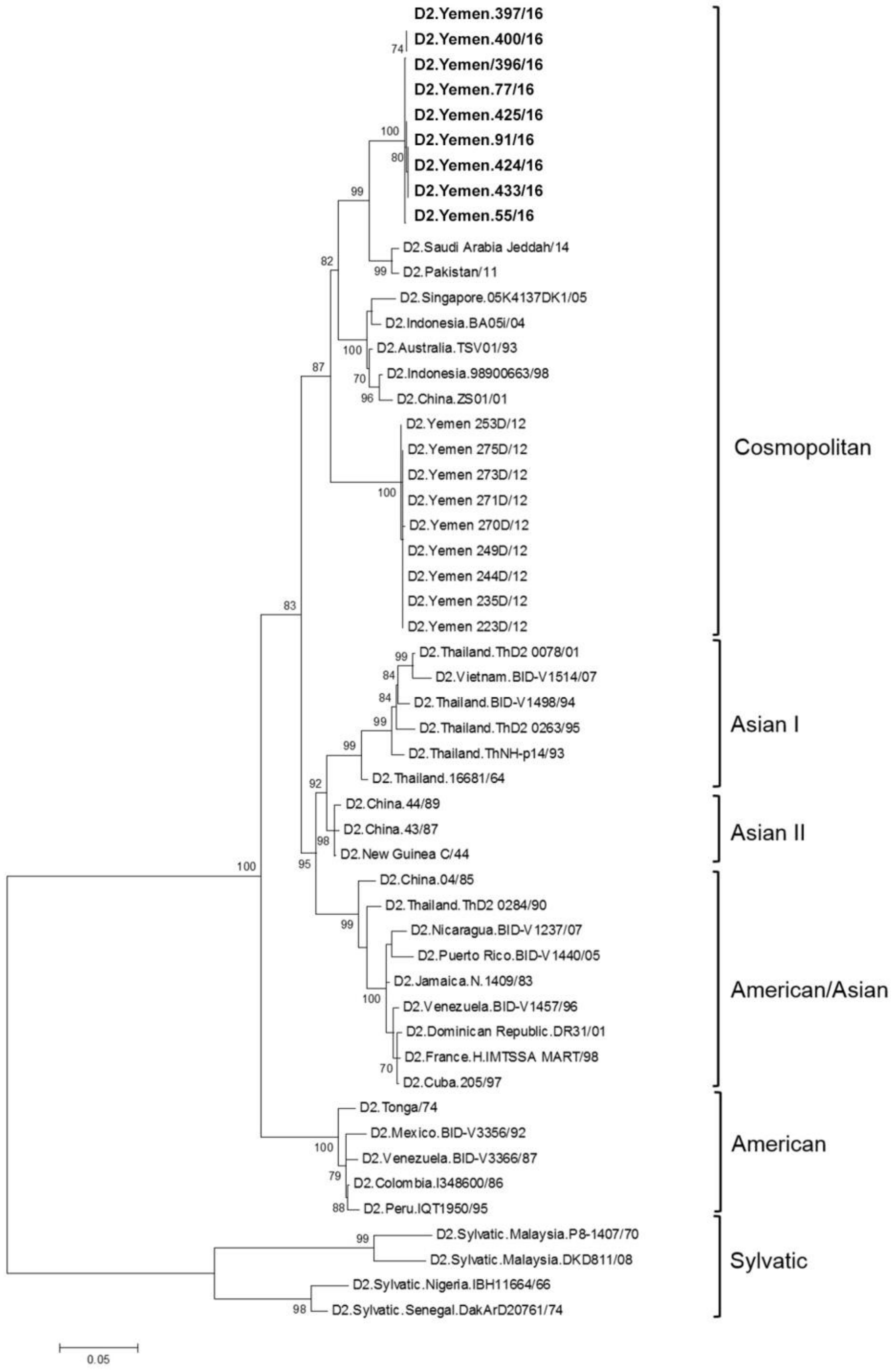

Appendix Figure 2. Maximum-likelihood phylogenetic tree of dengue virus type 2 (DENV-2) isolated in Taiz, Yemen, 2016. The tree was constructed by using nonstructural protein 1 gene sequences. DENV-2 strains identified in this study are indicated in bold. Numbers at nodes indicate bootstrap values (%) for 1,000 replicates. Only bootstrap values ≥70% are shown. Scale bar indicates nucleotide substitutions per site.
